# Modeling immune responses of cattle to *Mycobacterium bovis* using magnetic bioprinted granulomas

**DOI:** 10.1128/msphere.00595-25

**Published:** 2025-10-31

**Authors:** Gesa Krueger, Brahmaiah Meesaragandla, Lea Schultze, Katharina Pape, Ulrike Zedler, Gabriele Stooß, Shah Faisal, Kati Franzke, Stefanie A. Barth, Una Janke, Stefan H. E. Kaufmann, Björn Corleis, Mihaela Delcea, Anca Dorhoi

**Affiliations:** 1 Institute of Immunology, Friedrich-Loeffler-Institut, Federal Research Institute for Animal Health, Greifswald-Isle Riems, Germany; 2 Institute of Medical Biochemistry and Molecular Biology, University Medicine Greifswald 60634, Greifswald, Germany; 3 Institute of Biochemistry, University of Greifswald 26552, Greifswald, Germany; 4 Institute of Infectology, Friedrich-Loeffler-Institut, Federal Research Institute for Animal Health, Greifswald-Isle Riems, Germany; 5 Institute of Molecular Pathogenesis, Friedrich-Loeffler-Institut, Federal Research Institute for Animal Health, Jena, Germany; 6 Max Planck Institute for Infection Biology 28260, Berlin, Germany; 7 Max Planck Institute for Multidisciplinary Sciences 28282, Göttingen, Germany; 8 Charité-University Medicine Berlin, Berlin, Germany; 9 Faculty of Mathematics and Natural Sciences, University of Greifswald 26552, Greifswald, Germany; Washington University in St. Louis School of Medicine, St. Louis, Missouri, USA

**Keywords:** mycobacteria, tuberculosis, granuloma, cattle, 3D model, immunity

## Abstract

Tuberculosis (TB) remains a threat for human and livestock health. Mycobacteria causing TB are host-adapted pathogens that occasionally spill over into other species. *Mycobacterium bovis* causes bovine TB, a well-known zoonosis. *Mycobacterium tuberculosis* is adapted to humans and can occasionally trigger symptomatic infection in cattle. However, immunocompetent cattle are resistant to experimental infection with *M. tuberculosis*. Hallmarks of TB in susceptible hosts are organized multicellular tissue lesions termed granulomas. In the absence of suitable *in vitro* systems that enable investigations of bovine tuberculous granuloma, we developed a three-dimensional granuloma model using bovine leukocytes and magnetic cell labeling. This model was termed the *in vitro* granuloma-like structure (IVGLS). We generated stable IVGLS resembling TB granulomas at the innate stage, composed of macrophages, and at adaptive stages, containing lymphocytes in addition. *M. bovis* Bacillus Calmette-Guérin (BCG) replicated within IVGLS and triggered the progression of macrophages to foamy phenotypes. Within the IVGLS, the lymphocytes accelerated BCG-induced apoptotic cell death over time. IVGLS released abundant chemoattractants and Th1-associated cytokines and adopted a glycolytically polarized metabolism. Magnetic bioprinted bovine granulomas recapitulate features of TB granulomas and thus facilitate the study of immune responses to mycobacteria, including spatial and temporal mapping, as well as establishing precise cell death patterns within multicellular microenvironments. Deciphering protective immune responses within IVGLS can contribute to vaccine development for bovine TB, and elucidation of resistance mechanisms can facilitate the design of novel interventions for human TB.

**IMPORTANCE:**

Mycobacterial infections, including bovine tuberculosis (TB), have a profound impact on global health. This is exemplified by zoonotic TB in humans and animal TB, which is a life-threatening disease in livestock and wildlife. Mycobacteria cause the formation of granulomas, which significantly impact disease progression. Therefore, decoding granulomas is essential for an in-depth understanding of immune responses to mycobacteria. Conventional mouse models frequently fail to develop organized granulomas, and the procurement of samples from granulomatous lesions in cattle and humans is challenging, offering limited insights into the course of infection. Most *in vitro* TB research is confined to two-dimensional cell cultures, which neglect the spatial characteristics and cellular architecture of granulomas *in vivo*. To address this gap in knowledge, we have developed a novel multicellular *in vitro* model for TB. Our spheroid granuloma model, derived from bovine leukocytes using nanotechnologies, offers an adaptable platform for deciphering immune events within granulomas.

## INTRODUCTION

Mycobacterial infections caused by members of the *Mycobacterium tuberculosis* complex (MTC) have co-evolved with their hosts for thousands of years and, until today, have a major impact on public health (1–3). Despite genetic similarities of 99.9% in their 16S rRNA, members of the MTC differ substantially in their host tropism (4, 5). *Mycobacterium tuberculosis* (*Mtb*) is a human-adapted pathogen that has afflicted mankind for millennia, and tuberculosis (TB) remains the leading cause of death by a single infectious agent (1). In animals, *Mycobacterium bovis* causes bovine TB. Since common diagnostic procedures do not differentiate between *Mtb* and *M. bovis,* transmission of *M. bovis* to humans remains an underestimated risk in many TB-endemic areas (6). Although eradication programs decreased incidences of bovine TB in the past decades, bovine TB is still endemic in many countries, leading to significant economic losses (3, 7). Knowledge of the disease in cattle lags behind that in humans, leaving a significant gap in the understanding of immune reactions in the bovine host.

The pathological hallmark of TB in all species is the formation of granulomas. Mycobacteria are readily phagocytosed by alveolar macrophages after aerogenic infection and may translocate to the lung interstitium (8). In the course of infection, monocytes and macrophages are recruited, and the “core” of granulomas is formed (9). Once dendritic cells, neutrophils, and lymphocytes enter granulomas, they develop into organized structures containing multiple cell types (10–12). In this environment, macrophages undergo transformation into multinucleated giant cells, epithelioid macrophages, and foamy macrophages, which populate TB granulomas (10). TB outcome correlates with severity of the lesions, which range from solid to necrotizing “caseous” to successfully cleared “sterile” granulomas (13). In cattle, TB granulomas have been classified as stage I (initial), stage II (solid), stage III (minimal necrosis), and stage IV (necrosis and mineralization) based on observations from experimentally infected animals (14). Within these structures, B and T lymphocytes are initially scattered (stage I and II) and form the typical cuff in stage III/IV granulomas, similar to observations in developing human and non-human primate granulomas (15). The heterogeneity of granulomas and their zonation, including distinct immune cell topography (16), mirrors the complex host-mycobacteria interactions. Newly described microenvironments and cellular ecosystems highlight the complexity of the granuloma architecture (17, 18).

Animal models have contributed substantially to the understanding of immune responses to mycobacteria, but they infrequently reflect the histopathology of TB. Mice are not natural hosts of *Mtb* or *M. bovis* and largely lack human-like pathology. Non-human primates and livestock species develop pathology as seen in natural hosts, but experimental costs as well as ethical concerns pose limitations (19). Granuloma samples from natural hosts are difficult to obtain and only reflect a snapshot of the disease. Importantly, natural TB hosts differ in their defense mechanisms against mycobacteria. For instance, production of nitric oxide (NO), an important antimycobacterial agent, is virtually absent in human macrophages (20), while bovine macrophages do release NO (21), and bovine TB granulomas present abundant inducible nitric oxide reactivity, particularly prior to mineralization (22). In recent years, efforts have been made to develop spheroid models to account for the three-dimensionality of granulomas (23). Although granuloma fibrosis and caseation are still difficult to recapitulate, granuloma models enable investigation of diverse immune cell populations and their effects on granuloma architecture (24). Using *Mtb* antigen-coated beads, the appearance of multinucleated giant cells and epithelioid transformation of macrophages has been reported (25). Other approaches are based on collagen matrices or bio-electrospraying (26–31). 3D *in vitro* models that provide a hypoxic environment have been linked to a dormant-like state of *Mtb* that cannot be demonstrated in traditional cell cultures (27). For cattle, models for TB granulomas are rare (32).

We report a novel granuloma model made of bovine leukocytes. *In vitro* granuloma-like structures (IVGLS) were obtained using nanoparticles and therefore did not require a physical matrix. IVGLS were designed to reflect different stages of TB granulomas: innate IVGLS, made of macrophages, and mature IVGLS, containing lymphocytes in addition. Besides displaying cell transformation and glycolytic activity, IVGLS unveiled distinct spatial cell death patterns of macrophages and the release of type 1-associated cytokines and chemokines. Stable IVGLS recapitulate characteristic features unique to granulomas that are absent from conventional monolayer cell cultures, providing a practical framework for high-throughput granuloma research.

## MATERIALS AND METHODS

### Monocyte isolation and macrophage differentiation

Blood was drained by venepuncture from a cohort of four healthy, interferon-gamma release assay (IGRA)-negative Holstein cattle in tubes containing ethylenediaminetetraacetic acid (Sarstedt, Germany) and was subjected to peripheral blood mononuclear cell (PBMC) isolation. All steps were carried out at room temperature (RT) if not mentioned otherwise. The blood (diluted 1:1 with Dulbecco’s phosphate-buffered saline [DPBS]; Corning, NY, USA) was carefully layered onto Biocoll (density: 1.077 g/mL) (Bio&SELL, Germany) and centrifuged at 1,200 × *g* for 20 min with minimum acceleration and deceleration. Platelets were removed from the PBMC fraction by centrifugation at 100 × *g* for 10 min, and the resulting cell pellet was washed with DPBS at 400 × *g* for 10 min. Viability was assessed using erythrosin B (Roth, Germany). A total of 2.5 × 10^7^ PBMCs in 10 mL DPBS were seeded into a petri dish and incubated for 45 min at 38°C, 5% CO_2_ to allow monocytes to adhere. If T75 cell culture flasks were used, 5 × 10^7^ PBMCs were seeded in 10 mL DPBS. Non-adherent cells were collected, and adherent monocytes were washed twice with DPBS. Monocytes were incubated in 10 mL (or 15 mL for T75 cell culture flasks) Roswell Park Memorial Institute 1640 medium (RPMI) (Biowest, Nuaillé, France) supplemented with 10% fetal calf serum (FCS) (PANBiotech, Germany), 2 mM L-glutamine (Gibco, Thermo Fisher Scientific, Germany), 10 mM HEPES, and 50 µM β-mercaptoethanol (Sigma-Aldrich, Germany), referred to as complete RPMI (cRPMI), for 7 days at 38°C, 5% CO_2_ to differentiate into macrophages, whereby media was added after 4 days of incubation. Non-adherent cells were spun down (400 × *g*, 10 min) and cryopreserved using freezing media consisting of 90% FCS and 10% dimethyl sulfoxide (Sigma-Aldrich, Germany). Before the harvest of bovine monocyte-derived macrophages (bMDMs), a 24-well ultra-low-attachment plate (Thermo Scientific Nunclon Sphera, Germany) was incubated with RPMI for at least 30 min to reduce hydrophobic properties. Differentiated bMDMs were rinsed once with cold DPBS, and Accutase solution (Invitrogen, Thermo Fisher Scientific, Germany) was used for detachment. bMDMs were seeded at 2.5 × 10^5^ cells/500 µL cRPMI in each well and rested at 38°C, 5% CO_2_ overnight. For viability assessment, bMDMs were detached, stained with Zombie NIR dye, and acquired on BD Fortessa.

### Bacillus Calmette-Guérin (BCG) infection in ultra-low-attachment plates

*M. bovis* BCG Danish expressing mCherry or GFP (BCG mCherry, BCG mGFP) were used for all experiments (33). BCG was grown in Middlebrook 7H9 broth (BD Biosciences, USA) supplemented with 0.2% glycerol (Sigma-Aldrich), 0.05% Tween 80 (Sigma-Aldrich), and 10% Middlebrook albumin dextrose catalase enrichment (BD Biosciences). Cultures were maintained at 37°C, protected from light under shaking, and were used in logarithmic phase (OD_600_ = 0.3–0.8). The BCG suspension was centrifuged for 5 min at 18,000 × *g* 4°C and washed twice with DPBS before adjusting to achieve a multiplicity of infection (MOI) of 0.1. Infected bMDMs were incubated for 4 h at 38°C, 5% CO_2_, and subsequently, plates were centrifuged at 400 × *g* for 5 min to minimize cell loss prior to washing twice with cRPMI.

### Generation and characterization of nanoparticles

Individual nanoparticles (NPs) were synthesized separately before mixing and cell labeling. The following steps were performed at RT. Briefly, for the synthesis of citrate-coated iron oxide NPs (IO-NPs), equal amounts of 0.2 M iron(III) chloride (FeCl_3_, Sigma-Aldrich) and 0.5 M ammonium hydroxide (NH_4_OH, Sigma-Aldrich) were mixed under sonication to form colloidal iron(III) hydroxide (Fe(OH)_3_). Subsequently, 0.2 M iron(II) chloride (FeCl_2_, Sigma-Aldrich) was added at a ratio of 1:2 under sonication and poured into six volumes of 0.5 M NH_4_OH aqueous solution. This mixture was then stirred for 15 min to achieve magnetite coagulation. The colloid was then magnetically separated and washed twice with ultrapure water to remove all impurities (including NH_4_Cl). Sodium citrate (0.1 M, Alfa Aesar, USA) was slowly added under sonication to generate citrate-stabilized Fe_3_O_4_ NPs (IO-NPs). The colloid was washed again with ultrapure water, and the IO-NPs were dried completely before weight measurement. For poly-L-lysine (PLL) coating, 200 µL PLL (1 mg/mL, Merck) was added dropwise to 10 mL of citrate-IO-NPs (2.2 mg/mL) under stirring. NPs were magnetically separated and washed twice with ultrapure water. Finally, the mixture was sonicated for 5 min. For the synthesis of citrate-Au-NPs, 250 µL of 200 mM tetrachloroauric acid (HAuCl_4_, Alfa Aesar, USA) was added to 50 mL of 4 mM sodium citrate solution and heated up to 95°C under stirring for 15 min. The mixture was stored at 4°C before further use. NPs were freshly mixed before use (see “Generation of bovine IVGLS”). The formation of Au-NPs was characterized via UV-Vis absorption spectroscopy (Denovix DS-11 FX, Biozym Scientific, Germany). The hydrodynamic diameter and size distribution of NPs were determined using a Zetasizer Ultra (Malvern Panalytical, UK). Two batches of NPs with similar physicochemical characteristics were used throughout the study. Particle number per volume was assessed, and ratios were adjusted to guarantee similar composition and features of NPs independent of the manufacturing batch. NPs were checked for sterility on LB agar before use.

### Generation of bovine IVGLS

For comparative evaluation, we assembled naïve IVGLS containing uninfected bMDMs and IVGLS generated from BCG-infected bMDMs. This allowed discriminating the effect of BCG in the context of macrophage-derived 3D spheroids. Innate IVGLS consisting of infected bMDMs only and mature IVGLS, containing bMDMs and lymphocytes from the PBMC fraction, were obtained. Per well, 2.5 × 10^5^ bMDMs were labeled with 20 µL of a mixture containing PLL-coated IO-NPs and citrate Au-NPs. NPs were vortexed for 5 min and, depending on the batch of NPs, 750 µL or 615 µL DPBS was mixed with 200 µL or 137 µL IO-NPs and 250 µL Au-NPs before use. The NPs were added using a low-retention cut tip, and subsequently, the plate was maintained on a panning table for 30 min before incubating at 38°C, 5% CO_2_ overnight. The next day, a levitation magnet was applied above the cell culture plate. After 24 h of levitation, the magnet was placed underneath the culture plate in order to bioprint the preformed spheroids. The bioprinting magnet was kept for either 24 h or 96 h. After removing the magnet, the bioprinting magnet was only used when plates were moved from the incubator to prevent damage to the structures. In certain conditions, 2 days post-infection (dpi), 1 × 10^6^ non-adherent, autologous PBMCs (aPBMCs) in cRPMI were added to form mature IVGLS. aPBMCs were obtained by thawing the non-adherent fraction of the PBMC. Cells were resuspended in pre-warmed cRPMI supplemented with 1% penicillin and streptomycin. After washing, aPBMCs were rested in a six-well plate at 38°C, 5% CO_2_ overnight. Cells were resuspended at 5 × 10^6^ /mL in antibiotic-free cRPMI.

### Monolayer cultures of bMDM

bMDM and aPBMC for the designated “monolayer” were seeded in 24-well cell culture-treated plates (Costar Corning, Germany) at the same cell number and ratio of bMDM to aPBMC as for the IVGLS. No NPs, levitation, or bioprinting magnet were used. These cell culture conditions replicate the *in vitro* macrophage culture setup usually used in biomedical research.

### Morphometry of IVGLS

Light microscopy images of IVGLS were collected with a Nikon Eclipse TS2 inverted microscope with integrated camera. NIS-Elements software (“Basic Research” package) was used to measure the area of the IVGLS.

### Phenotyping of the PBMC fraction

The non-adherent PBMC fraction (aPBMC) gained during the monocyte isolation was characterized via flow cytometry. PBMCs were thawed and rested overnight as for the construction of the IVGLS. A total of 2 × 10^6^ cells were distributed into 96-well polypropylene plates and washed once with PBS, including a centrifugation step at 300 × *g* for 5 min at 4°C. Cells were stained with Zombie Red (1:250 in PBS; Biolegend, Germany) for 20 min at RT. After washing with PBS, samples were blocked with 50 µg/mL purified bovine IgG (Fortis live science, USA) for 10 min on ice. Samples were washed again with PBS, and primary antibodies (anti-CD3, clone: MM1A, Washington State University, USA, dilution 1:150; anti-CD4-Alexa Fluor 700, clone: CC8, Bio-Rad, Germany, dilution: 1:800; anti-TCR-d, clone: GB21A, Washington State University, USA, dilution: 1:100; anti-CD21-FITC, clone: CC21, Bio-Rad, Germany, dilution: 1:800) in FACS buffer (0.2% bovine serum albumin [BSA; Roth, Germany] in PBS) were added and incubated for 20 min on ice. Cells were washed twice with FACS buffer and blocked with 5% goat serum for 5 min. Secondary antibodies (anti-mouse IgG1-BV421, clone: RMG11 Biolegend, Germany, dilution 1:200; anti-mouse IgG2b-PE-Cy7, Biolegend, Germany, dilution: 1:400) in FACS buffer were added, and cells were further incubated for 20 min on ice. After washing, samples were resuspended in FACS buffer and immediately acquired on the BD FACSymphony A3 Cell Analyzer. Data analysis was done using FlowJo (version 10.9). Gates were set according to fluorescence minus one controls.

### Ultrastructural analysis of NP-labeled bMDM

bMDM cultivated in petri dishes for 7 days were harvested and rested overnight in a low-attachment plate before they were treated with NPs as previously described. A total of 1 × 10^7^ cells were embedded in 1.8% agarose dissolved in 0.1 M sodium-cacodylate buffer. Pre-fixation was done with 2.5% glutaraldehyde. Osmium tetroxide was used post-fixation to add density and contrast to the samples. Over 2 days, the embedding, including negative staining with uranyl acetate and dehydration using an ethanol serial dilution, was performed in the Lynx II embedding automat (Electron Microscopy Science Service). The glycid ether 100 infiltration solution was used to further enhance the contrast. For polymerization and block insertion into the capsule, a mix of glycid ether 100, methylnadic anhydride, dodecenyl succinic anhydride, and DMP-30 was used. Ultra-thin sections were cut using an ultramicrotome (Leica Microsystems, Germany) and placed on nickel nets. Sections were analyzed with a transmission electron microscope (Tecnai Spirit, FEI), and images were taken with an Eagle 2k CCD camera.

### T and B cell staining within the IVGLS

At 3 dpi, IVGLS were fixed with 4% paraformaldehyde (PFA, Sigma-Aldrich, Germany) for 1 h at RT, permeabilized with 0.1% Triton X-100 (Sigma-Aldrich, Germany), and blocked with a solution containing 0.2% BSA and 2% goat serum in PBS for an additional 1 h at RT. IVGLS were washed once with PBS while maintaining the bioprinting magnet applied. Primary antibodies (anti-CD3, clone: MM1A, Washington State University, USA, dilution 1:150; anti-CD79a; clone: HM47, BD Biosciences, Germany, 20 µL per IVGLS; anti-IBA1; polyclonal; Fujifilm; USA; dilution 1:500) were added in 0.1% Triton X-100, 0.2% BSA in PBS, and incubated overnight at 4°C. The IVGLS were washed twice with PBS, and secondary antibodies (goat anti-rabbit Alexa Fluor 488; Invitrogen, Germany, dilution: 1:500; anti-mouse IgG1 BV421, clone: RMG1-1, Biolegend, Germany) as well as SYTOX Deep Red nuclear stain (1:500, Thermo Fisher) were added in 0.1% Triton X-100 in PBS and incubated for 2 h at RT. Finally, IVGLS were washed twice with PBS and imaged with the CellInsight CX7 (Thermo Fisher) using the confocal mode and HCS software. Images were acquired with 20× magnification, 4 × 4 binning, and 35 steps with a step size of 5.185 µm. The image areas were automatically tiled by the HCS software of the confocal microscope, which resulted in dashed line patterns visible in the acquired images.

### Colony-forming unit (CFU) assay

CFU assays were performed at 3 and 7 dpi. To verify the number of phagocytosed BCG, supernatant and cell lysates were plated after 4 h of incubation. Cells and cell pellets were lysed with 0.05% Triton X-100 (Sigma-Aldrich, Germany), and serial dilutions were plated on Middlebrook 7H11 (Merck, Germany) agar base supplemented with 0.5% glycerol (Sigma-Aldrich, Germany), 10% Middlebrook oleic acid albumin dextrose catalase enrichment (BD Biosciences, USA), and hygromycin (Roth, Germany). CFU were enumerated after incubation at 37°C for at least 21 days.

### Lactate dehydrogenase (LDH) assay

LDH was measured in supernatants of IVGLS and monolayers using the CytoTox 96 Non-Radioactive Cytotoxicity Assay (PROMEGA, Germany) following vendor’s instructions.

### Viability assessment of whole IVGLS using high-content imaging

IVGLS were incubated with 8 µM Caspase 3/7 Green Detection Reagent (Thermo Fisher, Germany) for 1 h at 38°C, washed once with DPBS, and subsequently stained with Zombie NIR (1:100) (Biolegend, USA) and Hoechst 34580 (1:500) (Sigma-Aldrich, Germany) in PBS for 1 h at RT. After washing, IVGLS were fixed with 4% PFA for 1 h at RT. The bioprinting magnet was used during the entire staining procedure. Imaging was done with the CellInsight CX7 with confocal mode and the HCS software. Images were acquired with 20× magnification, 4 × 4 binning, and 35 steps with a step size of 5.185 µm. Data from images were set to thresholds of negative controls as well as validated using positive controls (Triton X-100, Camptothecin). Cell count was based on nucleus staining, and segmentation was based on nucleus signal. Cells at the borders between the fields of views and doublets (based on size) were excluded in order to maintain accuracy and avoid double counting. For imaging using the Leica THUNDER Dmi8 microscope, large volume computational clearing was applied within Leica Application Suite X. Images were acquired with 10× magnification, step size of 3.80 µm, and the number of steps was adjusted to the depth of IVGLS. 3D rendering was done based on maximum projection settings, and videos were saved with high-render settings.

### Evaluation of the neutral lipid content in IVGLS

IVGLS were dislodged by pipetting in DPBS. Cells from dislodged IVGLS were allowed to adhere to 0.01% poly-L-lysine (Sigma-Aldrich) treated coverslips for 30 min at 38°C, 5% CO_2_. IVGLS and monolayer samples were stained with 4 µg/mL BODIPY 493/503 (Invitrogen, Thermo Fisher, Germany) in DPBS for 30 min at RT and stained with Hoechst 34580 (Sigma-Aldrich) for 10 min, washed twice with DPBS, and fixed in 4% PFA for 20 min at RT before acquisition with CellInsight CX7. For manual evaluation of foamy cells, at least 100 cells were counted. Images were acquired with 20× magnification. Image processing was done using NIS-Elements (Nikon Eclipse TS2), HCS software (CellInsight CX7), and FIJI (ImageJ2).

For the evaluation of lipid bodies in intact IVGLS, aPBMCs were labeled with 5 µM SYTO 82 Orange nucleic acid stain (Thermo Fisher) in Hanks’ balanced salt solution without phenol red (Thermo Fisher, Germany) for 1 h before adding 1 × 10^6^ labeled aPBMCs to the respective IVGLS at 2 dpi. The next day, IVGLS were washed once with DPBS while the bioprinting magnet was applied. 8 µM BODIPY 493/503 and Hoechst 34580 in 200 µL PBS were added to IVGLS. After staining for 1 h at RT protected from light, IVGLS were washed once with DPBS and fixed with 4% PFA for 1 h. Finally, IVGLS were washed once with PBS and acquired on the CellInsight CX7 using confocal mode and HCS software. Staining time was reduced by half for the monolayers.

### Magnetic bead-based multiplex cytokine assay

Cytokine and chemokine levels in supernatants from IVGLS and monolayers were measured using the MILLIPLEX 15-plex bovine bead panel assay (Merck Millipore, Germany). The assay was performed according to the manufacturer’s instructions. Samples were acquired on a BioPlex System 200 microplate reader featuring BioPlex Manager software 4.0. Quality controls were validated with the values supplied by the manufacturer. At least 50 single beads per well were measured.

### Enzyme-linked immunosorbent assay (ELISA)

Dilution of supernatants was optimized before quantification using the ELISA for interleukin (IL)-8 (mabTech, Sweden). The assay was performed according to the manufacturer’s instructions.

### Metabolic flux measurements

Metabolic flux measurements were done with naïve or stimulated bMDMs. A total of 4 × 10^4^ cells were seeded in an XF96 cell culture microplate and left untreated or stimulated with 1 µg/mL lipopolysaccharide from *Escherichia coli* (LPS, InvivoGen, Germany), 1 µg/mL LPS in combination with 556 U/mL recombinant bovine interferon-γ (IFN-γ; R&D Systems, USA), irradiated *M. tuberculosis* H37Rv (BEI Resources, USA), and irradiated *M. bovis* AF 2122/97 (BEI Resources) at MOIs 3 and 10 for 24 h. Glycolysis and oxidative phosphorylation were determined using a Seahorse XFe96 Analyzer according to published protocols (34). Glucose (Agilent, USA), oligomycin (Agilent, USA), carbonyl cyanide 4-(trifluoromethoxy)phenylhydrazone (Sigma-Aldrich, Germany), and rotenone in combination with antimycin A (Sigma-Aldrich, Germany) were used to modulate the bioenergetics of previously stimulated cells. Assay medium containing Seahorse XF Base Medium (Agilent, USA), 2 mM L-glutamine (Gibco, Germany), 5 mM HEPES (Gibco, Germany), without glucose, phenol red, and sodium bicarbonate was freshly prepared. At the end of the assay, cells were fixed and stained with DAPI (Roth, Germany), acquired on CellInsight CX7, and cell numbers were used to normalize metabolic parameters.

### Lactate assay

The supernatants of the IVGLS and monolayer samples were collected at 1, 3, and 7 dpi and stored at −20°C until the assay was performed. Proteins were precipitated with 3% meta-phosphoric acid (Sigma-Aldrich, Germany) and spun down at 18,300 × *g*. Subsequently, 5 µL was transferred to a 96-well plate (Greiner, flat-bottom) and mixed with 100 µL master mix containing 27 mM NAD-free acid (Sigma-Aldrich, Germany) and 0.6 M glycine-hydrazine buffer (Sigma-Aldrich, Germany). As a start, a solution of 2 mg/mL LDH (Sigma-Aldrich, Germany) in 0.5 M glycine-hydrazine buffer was used. The samples were incubated in the dark for 2.5 h. Sodium lactate (Sigma-Aldrich, Germany) diluted in RPMI was used as a standard. Spectrophotometric measurements were performed with a TECAN Spark Reader at an absorbance of 340 nm.

### Griess assay

Fifty microliters of supernatant was transferred to a 96-well plate (Greiner, flat-bottom). Sodium nitrite solution (NaNO_2_ in distilled water, Merck, Germany) in RPMI 1640 was used as standard. Fifty microliters of Griess reagent (Sigma-Aldrich, Germany) was added to the standard and samples, followed by a 15 min incubation in the dark. Optical density was measured with the TECAN Spark Reader at 540 nm.

### Fluorescence-based metabolic assays

IVGLS or monolayer samples were infected with fluorescence-reporter BCG, depending on the metabolic stain, and tested in duplicates. When using MitoTracker Orange CMTMRos (Thermo Fisher, Germany), cells were infected with BCG GFP. Cells were starved for 2 h in RPMI 1640 then stained with 100 nM MitoTracker in cRPMI for 1 h in the dark at 38°C. After washing with PBS, IVGLS were disrupted by pipetting in PBS. Monolayer cells were detached using Accutase (Invitrogen, Germany). Two samples per condition were pooled, transferred to polystyrene tubes (Sarstedt), and spun down at 400 × *g* for 5 min. Zombie NIR (1:250, Thermo Fisher, Germany) was used as a viability marker in PBS. After washing, samples were fixed with 4% PFA for 30 min at RT, acquired on a BD Symphony A3 or LSR Fortessa X20 flow cytometer, and analyzed using FlowJo software (version 10.8.).

### Statistical analysis

Statistical analyses were performed using GraphPad Prism (version 9.3.1). If feasible, data were tested for normality using the Shapiro-Wilk test and are presented as biological replicates. One-way ANOVA with Tukey’s test was used for the IVGLS evaluation. For the analysis of IVGLS vs monolayer and IVGLS at different time points, two-way ANOVA with Dunnett’s multiple comparison test was applied. A *P*-value of less than 0.05 was considered significant.

## RESULTS

### Generation of stable bovine IVGLS from mycobacteria-infected bMDM and autologous lymphocytes

Macrophages are bona fide host cells for mycobacteria and represent the minimal cellular ecosystem of granulomas. Prior to the generation of IVGLS, we evaluated culture conditions enabling differentiation of large numbers of viable bMDM from PBMCs. Since formation of the spheroids requires monocyte isolation and magnetic labeling (35), isolation procedures based on nanobeads were excluded. We evaluated bMDM yields after cultivation of the adherent monocyte fraction from PBMCs on variable substrates, notably plastic petri dishes and cell culture flasks. We avoided growth factors that polarize macrophages to restrict signaling interference during subsequent spheroid formation. Differentiated cells showed typical macrophage-like morphology under both culture conditions (Fig. S1A). The bMDM presented amoeboid morphology and often elongated spindle-like shape. In line with cell densities observed by microscopy, we recovered higher numbers of bMDM upon differentiation in petri dishes (Fig. S1B), and these showed excellent viability (Fig. S1C). The bMDM released NO upon stimulation by LPS and IFN-γ (Fig. S1D) and activated glycolysis upon bacterial stimulation (Fig. S1E).

**Fig 1:**
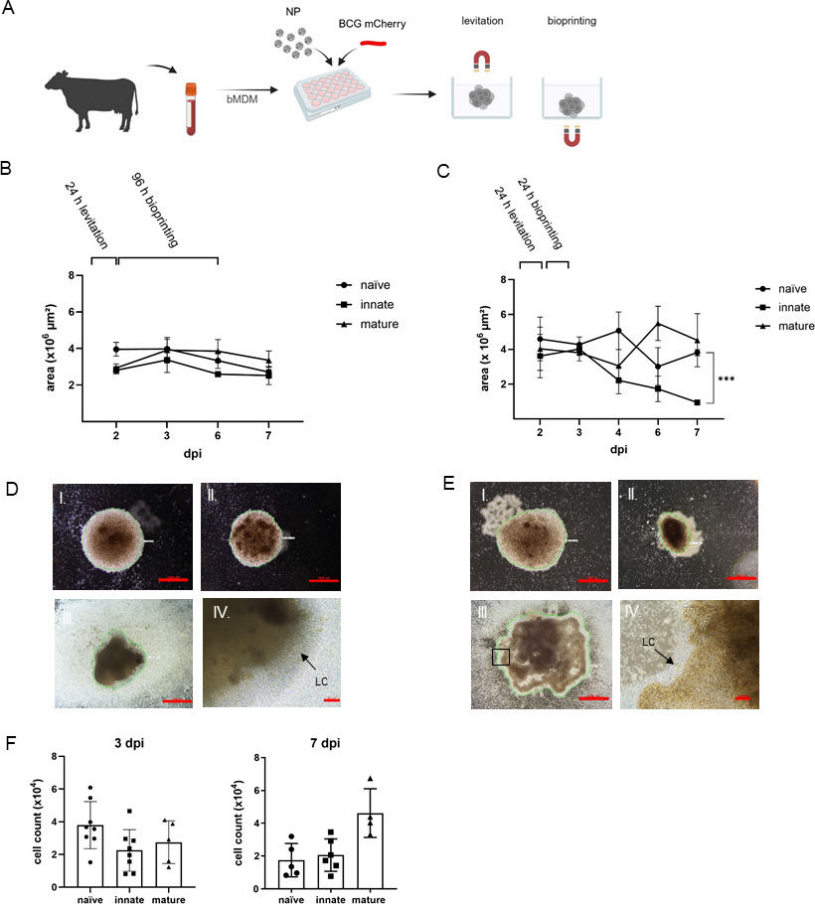
Features of bovine IVGLS obtained from BCG-infected bovine monocyte-derived macrophages. (**A**) Workflow used for generation of the IVGLS. (**B**) Size of IVGLS magnetic bioprinted for 96 h (*n* = 6). (**C**) Size of IVGLS magnetic bioprinted for 24 h (*n* = 4). Data show mean ± SD and was analyzed by one-way ANOVA with Tukey’s multiple comparison, ***P < 0.001. (**D**) Appearance of IVGLS obtained as in panel B was evaluated by wide-field microscopy over 1 week. Representative images of (I) naïve, (II) innate, and (III) mature IVGLS displayed with 2× magnification; scale bar: 1 mm; (IV) lymphocytic cuff (LC) of (III) with 10× magnification; scale bar: 100 µm. Green line indicates area measurement. (**E**) Representative images of (I) naïve, (II) innate, and (III) mature IVGLS obtained as in panel **C**, displayed with 2× magnification. Green line indicates area measurement. (**F**) Numbers of cells contained by IVGLS at 3 days and 7 days post-infection were evaluated using high-content microscopy with a confocal module. Bar plots show mean ± SD of IVGLS from individual donors (3 dpi: naïve *n* = 8, innate *n* = 8, mature *n* = 5; 7 dpi: naïve *n* = 5, innate *n* = 6, mature *n* = 4). Autologous PBMCs were added at 2 dpi, after the levitation period.

**Fig 2:**
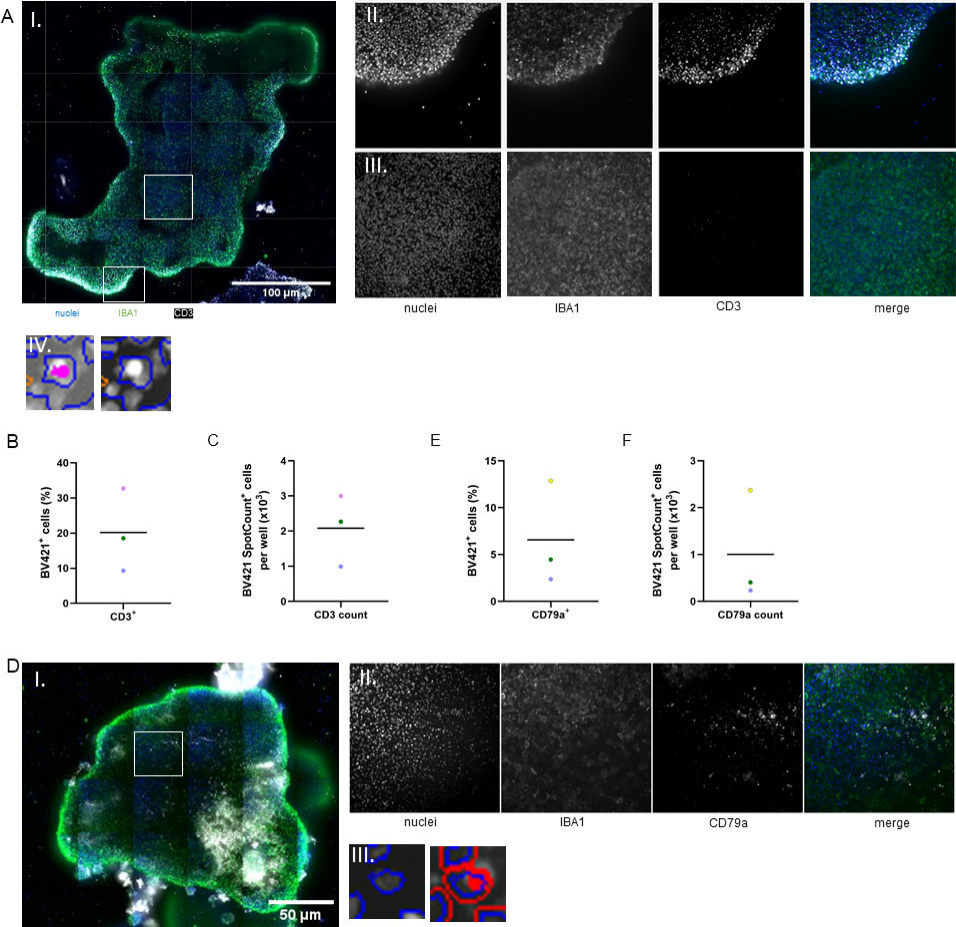
Autologous T cells form a cuff around bMDM in the IVGLS. (**A**) (I) Whole IVGLS stained for CD3 (T cells, white) and IBA1 (macrophages, green). Regions of interest (ROI) at the edge (II) and in the core (III) are indicated and are shown in higher magnification with split separate channels. Cell mask of a CD3 spot count^+^ cell is shown (IV). (**B**) Percentage of CD3^+^ cells in one IVGLS, mean indicated, color code depicts data from different individuals. (**C**) Absolute numbers of CD3 spot count^+^ cells, mean indicated, color code depicts data from different animals. (**D**) (I) Whole IVGLS stained for intracellular CD79a (B cells, white) and IBA1 (macrophages, green), ROI (II) is indicated and shown in higher magnification with split separate channels. (III) Shows the cell mask of a CD79a spot count^+^ cell. (**E**) Percentage of CD79a^+^ cells in one IVGLS, mean indicated, color code depicts data from different animals. (**F**) Absolute numbers of CD79a spot count^+^ cells, mean indicated, color code depicts data from different animals.

For comparative evaluation, we generated naïve IVGLS containing uninfected bMDM along with IVGLS assembled from BCG-infected bMDM. Innate or incipient-IVGLS consisting of infected bMDM reflect an early stage of TB granuloma. Mature, adaptive granulomas contain lymphocytes in addition, and these were generated by adding aPBMCs early during bioprinting. The composition of lymphocytes in the aPBMC fraction (Fig. S2) was typical for cattle PBMCs and mainly comprised T cells, especially γδ T cells, which made up to 52% of the T cell compartment (36). The 3D granuloma-resembling structures were hence termed naïve, innate, and mature IVGLS. The IVGLS generation workflow envisaged labeling of the BCG-infected bMDM with NPs, magnetic levitation at the air-liquid interface, eventual addition of aPBMC, and finally magnetic bioprinting of the spheroids (Fig. 1A). Ultrastructural analysis of bMDM treated with NPs showed presence of the NPs within the cell and at the membrane level, and no impact of the NPs on the overall ultrastructure of the macrophages (Fig. S3). bMDM showed a vacuole-rich phenotype, irrespective of NP treatment. Different bioprinting times were tested to optimize stability of the spheroids (Fig. 1B and C). Irrespective of the bioprinting time, only one spheroid per cavity was formed. Levitation for 24 h followed by 96 h bioprinting resulted in generation of stable and equally round-shaped IVGLS (Fig. 1B and D). A shorter bioprinting resulted in unstable IVGLS of variable sizes (Fig. 1C). The innate structures became smaller and denser over time (Fig. 1C). Since IVGLS showed appreciable variation in size and considering that the stability of the structures is critical for monitoring events within, we extended the bioprinting time. Mature IVGLS developed a well-formed cellular cuff upon addition of aPBMCs surrounding the core of infected bMDM (Fig. 1D and E IV.). The areas of mature and naïve IVGLS were comparable despite the lymphocytic cuff of the mature IVGLS (Fig. 1B and C), suggesting that dynamics, viability, and biomechanics of cells within spheroids containing mycobacteria were changed. Extending the levitation time did not affect the formation of the lymphocytic cuff (Fig. S4A and B). Evaluation of cell numbers in IVGLS using high-content microscopy, notably without disruption of the spheroids, confirmed that mature IVGLS contained more cells after 1 week, indicating accumulation of unlabeled lymphocytes to the BCG-infected spheroid core (Fig. 1F). In more detail, staining for CD3-positive T cells revealed their distribution at the edges of the IVGLS, whereas the core region was sparsely populated with T cells (Fig. 2A). At 3 dpi, the lymphocytes made up a considerable part of the mature IVGLS, reaching up to 32% frequencies, albeit with considerable inter-individual differences (Fig. 2B and C). B cells, identified as intracellular CD79a-positive cells, were present at lower frequencies (Fig. 2D through F). When multiple B cells were present, clumping of these cells at the periphery was observed (Fig. 2D). Despite variable cell numbers, IVGLS obtained subsequent longer bioprinting remained comparable in size. Magnetically labeled IVGLS were larger, up to 1 mm in diameter (Fig. 1B), than other *in vitro* spheroids (30, 32, 37) which are 150 µm–400 µm in size. Using unpolarized bMDM, we generated IVGLS with 3D structure, which resemble granuloma architecture and mimic different stages. Longer bioprinting time improved the stability of the IVGLS.

### Prolonged bioprinting facilitates mycobacterial growth within IVGLS

**Fig 3:**
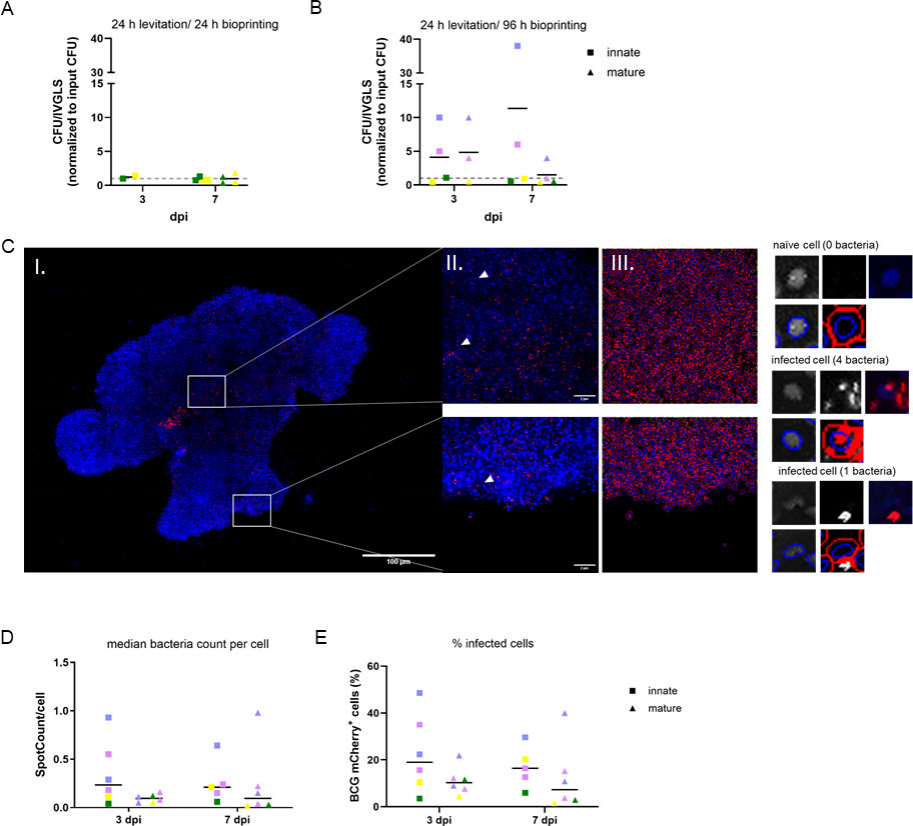
Stable IVGLS favor BCG replication. Bacterial burdens in IVGLS bioprinted for (**A**) 24 h or (**B**) 96 h. CFU values were normalized to the bacterial input at the IVGLS generation start (gray dotted line), each symbol denotes an individual donor indicated by color code, measured in duplicates, mean is indicated. (**C**) Innate IVGLS bioprinted for 96 h with (I) maximum intensity projection of whole innate IVGLS generated from 35 stacks with 5.185 µm steps. Staining visualizing nuclei (blue), BCG mCherry (red), scale bar: 100 µm. (II) White rectangle shows the regions of interest (ROI), arrowheads indicate infected cells, scale bar: 60 µm. (III) ROI (red lines), defined as circular cell mask around nucleus staining signal. (Right) Single-cell resolution shows nucleus, BCG mCherry, and their respective cell mask of naïve and infected cells. (**D**) Mean bacteria count per cell defined as spot counts in red channel (BCG mCherry) per ROI around nucleus. (**E**) Percentages of BCG-infected cells defined as spot count-positive cells. Panels **D and E** were evaluated in single, whole IVGLS bioprinted for 96 h at 3 and 7 dpi by high-content imaging, a minimum of 1,000 cells in the spheroid were evaluated, each symbol denotes an IVGLS from an individual donor indicated by color code, means are shown (*n* = 5–6).

To evaluate whether variation in size and stability alters mycobacterial replication within the IVGLS, the BCG load was determined over time by CFU assays and by high-content microscopy using fluorophore-tagged BCG. While mycobacterial growth was restricted in IVGLS bioprinted for 24 h (Fig. 3A), bacteria grew in innate and mature IVGLS bioprinted for 96 h. Thus, prolonged bioprinting improved stability and facilitated mycobacterial replication within the spheroid (Fig. 3B). This is in contrast to cell culture monolayers, where we could not detect BCG replication (Fig. S5). BCG-infected cells were distributed over the whole IVGLS (Fig. 3C). In line with the low MOI used, analysis of IVGLS generated with fluorescent reporter BCG unveiled few infected cells and comparable infectivity and mean infection rate in innate versus mature IVGLS (Fig. 3C through E). Thus, mycobacteria replicate within IVGLS and stable 3D spheroids offer BCG a replication advantage.

### Macrophages in mature IVGLS undergo cell death

**Fig 4:**
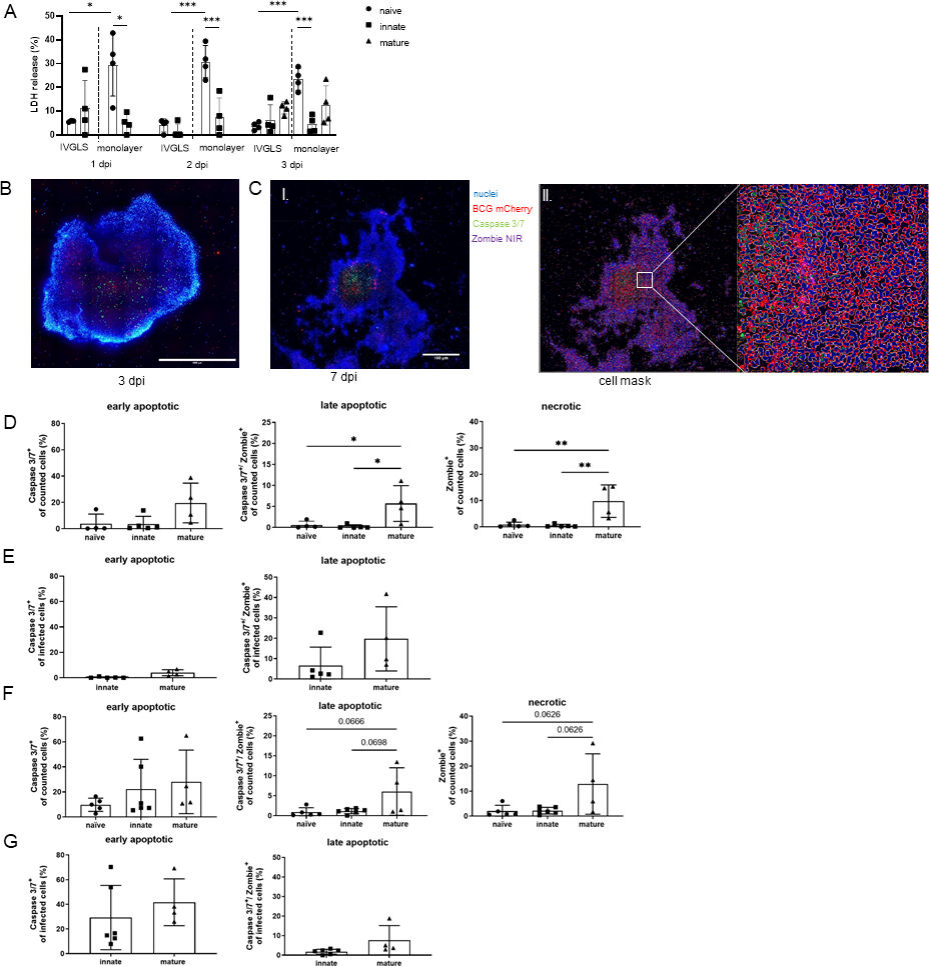
Macrophages undergo apoptosis in BCG-containing IVGLS. (**A**) LDH release of naïve, innate, and mature IVGLS or monolayers measured at 1, 2, and 3 dpi (*n* = 4). (**B**) Maximum intensity projection of whole innate IVGLS generated from 35 stacks with 5.185 µm steps at 3 dpi. Staining visualizing nuclei (blue), BCG mCherry (red), caspase 3/7^+^ cells (green), and Zombie NIR^+^ cells (purple), scale bar: 1 mm. (**C**) (I) Innate IVGLS at 7 dpi shown as in panel **B**, scale bar: 100 µm. (II, III) Cell mask (red line) was applied to identify cells containing BCG mCherry (red), Caspase 3/7 (green), and Zombie NIR (purple) spot count pattern. (**D**) Cell death patterns of all cells within IVGLS at 3 dpi. (**E**) Early and late apoptotic cells in BCG mCherry-infected population of IVGLS at 3 dpi (*n* = 4–5). (**F**) Cell death patterns in whole IVGLS at 7 dpi. (**G**) Early and late apoptotic cells in BCG mCherry-infected population of IVGLS at 7 dpi (*n* = 4–6). Early apoptotic (caspase 3/7^+^), late apoptotic (caspase 3/7^+^/ Zombie NIR^+^), and necrotic (Zombie NIR^+^) cells were evaluated in images from 35 stacks with a step size of 5.185 µm, a minimum of 1,000 cells in the spheroid were evaluated. Bar plots show mean ± SD, symbols represent individual donors. One-way ANOVA with Tukey’s multiple comparison test, *P < 0.05, **P < 0.01.

We observed discrepancies related to size and cell content in IVGLS. Based on these findings and considering that within granulomas, gradients of oxygen, nutrients, and antimycobacterial molecules can impact host cell viability (38), we investigated cell death patterns in IVGLS. Measuring LDH release, we observed that for IVGLS, viability was increased compared to monolayer cultures, and this was obvious for the naïve group (Fig. 4A). We further employed confocal high-content imaging to analyze the entire IVGLS, as dislodging could alter cell membrane integrity (Fig. 4B and C). Infected macrophages could be filtered, which enabled distinction of bystander effects within IVGLS from direct mycobacterial infection. We confirmed that within naïve IVGLS, macrophages remain largely viable for 1 week after initiating the 3D culture. Necrotic and late apoptotic death rates were below 5% in naïve IVGLS up to 1 week (Fig. 4D and F), indicating negligible cell stress by the magnetic labeling. Innate IVGLS showed increasing apoptosis over time, which was partially associated with the BCG infection status (Fig. 4E and G). Of note, more apoptotic cells were detected within the core of the IVGLS than in the outer rim (Video S1; Fig. 4B and C). The highest frequencies of late apoptotic and necrotic cells were observed in mature IVGLS (Fig. 4D and F). Most of the late apoptotic cells within IVGLS were not BCG-infected. Overall, these results indicate low levels of necrosis and moderate levels of apoptosis, which leave stability of IVGLS unchanged. Increased cell death in mature IVGLS may explain their reduced size despite the higher cell content and a tendency for lower CFU in mature IVGLS due to restricted replication niches for mycobacteria.

### Macrophages within IVGLS acquire foamy cell phenotype

**Fig 5:**
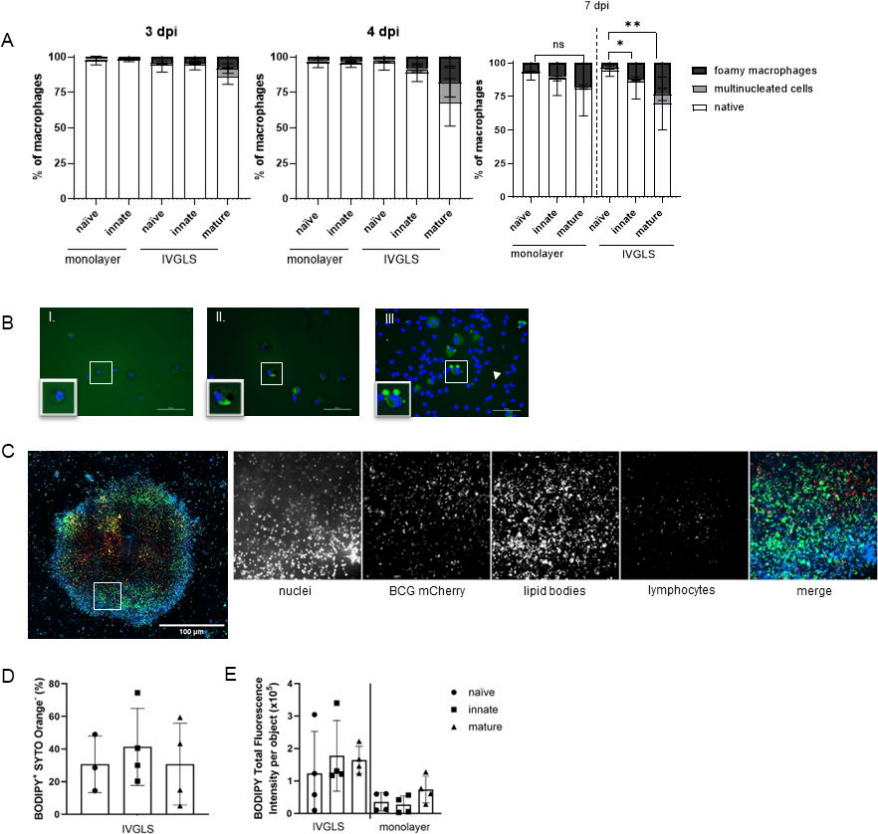
IVGLS promotes the transformation of macrophages into foamy cells. (**A**) Cellular composition of cells dislodged from IVGLS and monolayers was determined upon staining for lipid bodies (BODIPY 493/503), nuclei (DAPI) at 3 dpi (monolayer *n* = 4, IVGLS *n* = 6), 4 dpi (*n* = 4), and 7 dpi (*n* = 6). A total of 100 cells were manually counted using a Nikon Eclipse TS2 inverted microscope. Data show mean ± SD and were analyzed by two-way ANOVA with Dunnett’s multiple comparison test, *P < 0.05, **P < 0.01 refers to the foamy macrophage counts. (**B**) Representative images of foamy cells from different IVGLS, nuclei (blue), and lipid bodies (green puncta). (I) Macrophages from naïve IVGLS, (II) innate IVGLS and (III) macrophages and aPBMCs (arrowhead) from mature IVGLS. Region of interest showing a foamy macrophage. Overlays done with NIS-Elements. Scale bar, 50 µm. (**C**) Representative image of IVGLS stained for neutral lipids at 3 dpi. Staining visualizing nucleus (Hoechst 34580, blue), BCG mCherry (red), lipid bodies (BODIPY 493/503, green), and lymphocytes (SYTO Orange, yellow). White rectangle indicates regions of interest. (**D, E**) Lipid load quantified in whole IVGLS or monolayer 3 dpi by high-content imaging. (**D**) Percentage of foamy phenotype (BODIPY^+^) from all macrophages (SYTO Orange^−^) (*n* = 3-4). (**E**) Total fluorescence intensity per cell from various entire IVGLS and monolayer. Data show mean ± SD, and symbols indicate individual donors (*n* = 4).

Since macrophage transformation toward foamy cells is a hallmark of TB granulomas (39), we investigated their phenotype in IVGLS and traditional monolayers. Stable IVGLS accumulated foamy cells over time, shown in single cells from dislodged IVGLS as well as in whole IVGLS (Fig. 5). The BODIPY staining pattern revealed striking differences in neutral lipid abundances between cells dislodged from various IVGLS (Fig. 5A; Fig. S6), suggesting that the composition of the IVGLS affects macrophage metabolism. The most pronounced effect was observed after addition of lymphocytes (Fig. 5A and B). While macrophages from innate IVGLS mostly contain single lipid bodies in their cytosol, macrophages from mature IVGLS presented significantly more and larger lipid bodies (Fig. 5B; Fig. S6). Manual quantification indicated a significant difference of foam cell transformation between naïve and mature IVGLS, which started already at 4 dpi (Fig. 5A). Limited progression to polyploidy was detected upon prolonged incubation (Fig. 5A). The automated quantification of the fluorescence intensity levels revealed a trend toward higher lipid loads in IVGLS compared to monolayers at 3 dpi (Fig. S6B and C). The assessment of the entire IVGLS using high-content confocal microscopy showed high percentages of foamy macrophages, indicating that such fragile cells may be lost during IVGLS processing (Fig. 5C through E). Thus, IVGLS provides a microenvironment that enables the progression of macrophages toward foamy cells.

### Mycobacteria-containing IVGLS release abundant chemokines and cytokines

**Fig 6:**
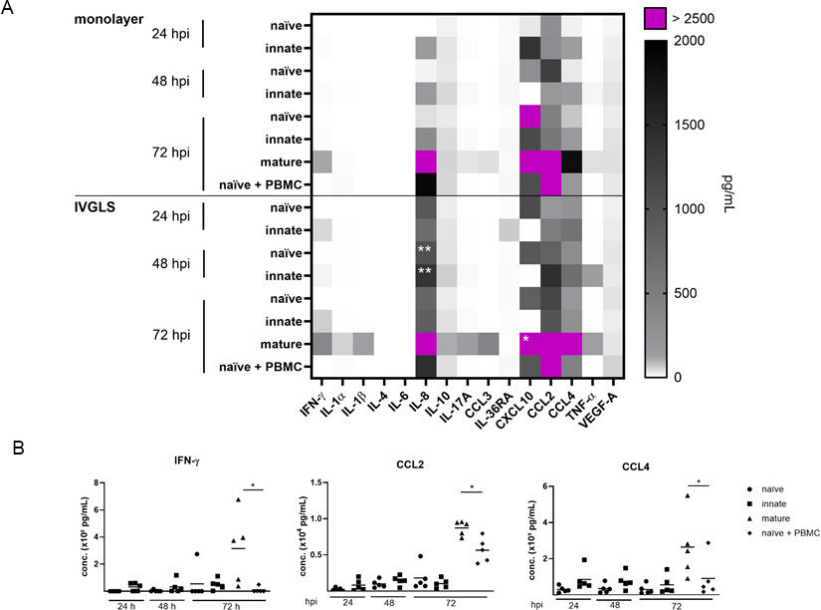
IVGLS secrete abundant chemokines upon mycobacterial infection. (**A**) Levels of cytokines, chemokines, and growth factors in supernatants of IVGLS and bMDM monolayers at 1, 2, and 3 dpi were determined by bead-based multiplex assays. Autologous PBMCs were added at 2 dpi. Heatmap integrates data from 4 to 5 donor cattle for each condition, data were analyzed with multiple paired *t*-test (IVGLS: *n* = 5; monolayer naïve, monolayer innate: *n* = 4; monolayer 72 h mature, naive + PBMC: *n* = 5). *P < 0.05, **P < 0.01. (**B**) IFN-γ, CCL2, and CCL4 were determined as in panel **A**, showing differences between various IVGLS (*n* = 5). Data show mean ± SD, one-way ANOVA with Tukey’s multiple comparison test, *P < 0.05.

Cell-free supernatants were collected at different time points post-infection in order to evaluate secretory features of different IVGLS in comparison to monolayers (Fig. 6A; Table S1). Cytokines of the IL-1 family, including IL-1α, IL-1β, and IL-36RA, were present at very low levels, substantiating that the used NPs did not cause inflammasome activation as reported for silica or titanium NPs (40). Moreover, low IL-6 levels in IVGLS confirmed that stress responses were not induced by labeling of the cells and/or by bioprinting. We detected higher concentrations of specific chemokines in IVGLS compared to monolayers. Significantly more CXCL10 and IL-8 were present in mature IVGLS compared to traditional cell culture systems (Fig. 6A; Fig. S7). Mature IVGLS also released abundant CCL2 and CCL4 (Fig. 6B), indicating regulation or release by lymphocytes. The multicellular composition of the mature IVGLS also contributed to an elevated release of IFN-γ in mature structures at 72 h post-infection. In sum, IVGLS released chemokines and Th-1-associated cytokines in higher abundance.

### Immunometabolism of IVGLS is largely directed toward glycolysis

**Fig 7:**
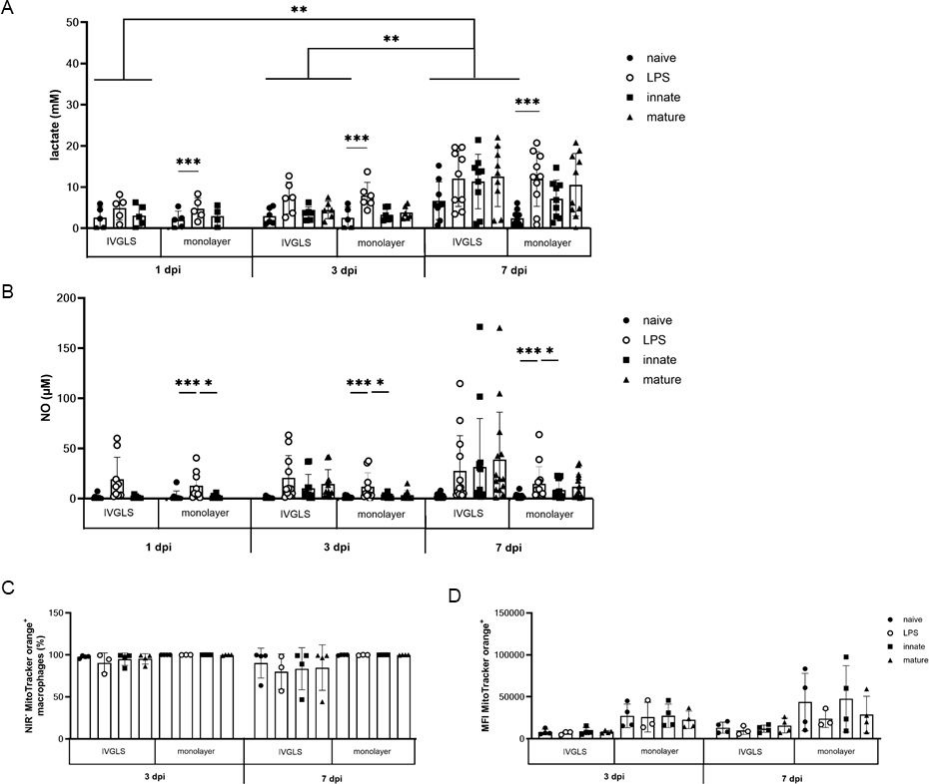
Metabolic rewiring of immune cells in IVGLS progresses primarily towards glycolysis. (**A**) Lactate (*n* = 10) and (**B**) NO (*n* = 14) concentrations in supernatants of IVGLS and monolayer samples. Symbols indicate mean from three technical replicates and show data from individual donors. (**C**) Macrophages dislodged from IVGLS or detached from monolayers were evaluated at 3 and 7 dpi using flow cytometry. (**D**) Mean fluorescence intensity of MitoTracker Orange in macrophages from IVGLS and monolayers at 3 and 7 dpi (*n* = 4; LPS *n* = 3). Data show mean ± SD from individual donors. Statistics calculated with two-way ANOVA with Tukey’s multiple comparison test, *P < 0.05, **P < 0.01, ***P < 0.001.

Metabolic changes accompany TB granuloma formation and have been reported for mycobacteria-infected macrophages (16, 41). We compared glycolysis and oxidative phosphorylation in IVGLS and macrophage monolayers. NO and lactate were measured as a proxy for glycolysis (34). Lactate levels increased significantly over 1 week in BCG-IVGLS and reached levels recorded for LPS-treated IVGLS (Fig. 7A). These kinetics were not statistically significant in monolayer cell cultures. NO levels in IVGLS tend to increase in comparison to monolayers (Fig. 7B), with considerably more abundant NO in supernatants from IVGLS of certain donors. As an indirect measurement of oxidative phosphorylation, we evaluated active mitochondria (34) and observed a decreased signal in IVGLS over time, but not in monolayers (Fig. 7; Fig. S8). Notably, even frequencies of macrophages containing active mitochondria were decreased over time within IVGLS (Fig. 7C). IVGLS are largely prone to glycolysis and thus recapitulate metabolic features of TB granulomas *in vivo*.

## DISCUSSION

The TB granulomas are heterogeneous and present a distinct cellular and metabolic zonation (18). Recent advances in spatial biology of human and non-human primate lesions emphasize the importance of microenvironments within granulomas for mycobacterial control and TB outcome (17, 42). Despite the economic impact of bovine TB outbreaks worldwide (3, 7), there is a knowledge gap related to granuloma architecture, cellular topography, and zonations in cattle. Here, we established a magnetic bioprinted *in vitro* granuloma-like model and conducted an in-depth characterization of BCG-IVGLS over time. As granulomas of different stages are present in individual patients (43) and range from solid to necrotic stages in cattle (14), we modeled IVGLS to mirror variability in cellular composition and structure. Stable bovine IVGLS recapitulated characteristics unique to granulomas that are absent from conventional monolayer cell cultures, providing a practical framework for TB granuloma research in cattle and other natural hosts.

A challenge in TB granuloma research and, more generally, in multicellular spheroid biology is represented by reproducibility and stability. In our study, extended bioprinting times in a magnetic field resulted in stable bovine IVGLS. Of note, the structures remained stable after magnet removal, suggesting that initial cell-cell contact is important to facilitate spheroid formation. E-cadherins drive macrophage epithelialization and play an important role in granuloma formation in humans, mice, and zebrafish (44). Macrophage-specific downregulation of E-cadherin expression leads to blockade of granuloma formation (44). We did not employ any growth factors during macrophage differentiation, but cannot exclude that cytokine release upon BCG infection may contribute to macrophage clustering via E-cadherin regulation. The extracellular matrix (ECM)-free approach enabled us to add additional immune cells at any time and provides a high degree of flexibility with regard to the IVGLS cellular landscape. Other models of TB granulomas employ ECM such as collagen-fibronectin (27–29) or alginate-collagen for the generation of microspheres or spheroids (30, 31). These restrict the option to add further immune cells at a later time point and alter the biomechanics by encasing the spheroids. Higher stiffness, such as conferred by plastic culture conditions, can increase PIEZO1 signaling and activate NF-κB and STAT6 signaling pathways, mediating pro-inflammatory phenotypes (45). Collagen-rich environments of 3D models, on the other hand, can favor immunosuppressive features in macrophages (46). The NPs used in this study harbor the potential to be functionalized, which could facilitate future studies on cell dynamics within the IVGLS. Magnetic cell levitation has been used in a patient-derived model of human TB and offers the potential to accelerate future development of host-directed therapies for TB (47). For cattle, IVGLS should help to understand granuloma biology in a natural host for mycobacteria and inform vaccine discovery using *ex vivo* testing methods.

The use of nanotechnologies enabled the generation of large spheroids comparable in size to organoids (48). Increasing the size of spheroids leads to greater gradients of nutrients and, especially, oxygen (49, 50). The hypoxic zone is a key feature of *in vivo* TB granulomas (16) and is only detected in certain experimental TB models such as rabbits, guinea pigs, and non-human primates (38). We speculate that hypoxic niches are present in the IVGLS, and that this physical factor is critical, as it alters the biology of the host cells (51) and that of mycobacteria (52). Hypoxia in TB granulomas is accompanied by glycolysis, which leads to the accumulation of glycolytic by-products such as lactate (53). Abundant lactate was detected in IVGLS, supporting a bias toward glycolysis. Nevertheless, the presence of hypoxic niches within the IVGLS requires further validation.

The increase of necrotic cells in IVGLS, including lymphocytes, points to intra-granulomatous cell death, whereas overall viability, including naïve macrophages, was increased in IVGLS. Albeit part of necrotic cells can be accounted for by aPBMCs due to freeze-and-thaw procedures despite resting before addition to the IVGLS, infected cells, primarily macrophages, also showed necrosis markers. Interestingly, we detected increased apoptosis in innate and mature IVGLS, and addition of lymphocytes accelerated apoptosis. Cell death within the granuloma is a defining event for stage classification *in vivo* (14). With one-third of apoptotic cells being infected, this may serve elimination of BCG. Apoptosis of bystander macrophages could restrict the replication niches within the IVGLS. The presence of lymphocytes in the mature IVGLS could favor mycobacterial clearance as we detected a trend toward reduced bacillary burdens in mature IVGLS. We observed significant variability in CFU enumerated for IVGLS generated from individual donor cattle. Interestingly, specifically early during bovine TB, a heterogeneous and variable bacterial load is noted for individual granulomas (54), which could indicate dynamic events that IVGLS likely recapitulate *ex vivo*. Several T cell subsets such as αβ CD4^+^, CD8^+^, and γδ T cells, which are present in high numbers in cattle, are a source of IFN-γ, a well-known effector molecule for mycobacterial control (55, 56). This type 1 cytokine was indeed present in mature IVGLS, as well as the interferon-inducible chemokine CXCL10. IVGLS may thus be harnessed for studying the contributions of various T cell subsets to mycobacterial control in multicellular systems. IFN-γ causes apoptotic death of *Mtb*-infected murine macrophages (57), and in murine lungs, complex regulation of apoptotic cell death by tumor necrosis factor (TNF) was reported (58). Since we observed heightened cell death also in bystander macrophages, it will be of interest to establish the molecular pathways governing apoptosis and necrosis in IVGLS in spatial context. IFN-γ priming of bystander cells could be a protective *cis* or *trans* effect, which may require cell-cell contact, or even act at a distance. Furthermore, the effect of xenophagy in macrophages of the IVGLS could be studied on the cell level with high-content technologies.

Since the formation of the lymphocytic cuff is guided by chemokines, we hypothesized that the 3D structure is richer in chemokines. Indeed, mature IVGLS released more abundant CCL2 and CCL4 compared to respective controls. Cytokine assessment further revealed high levels of IL-8 in IVGLS. IL-8 is a neutrophil attractant, and neutrophil recruitment modulates the fate of TB granulomas *in vivo* (11). Although neutrophils were not included in this study, neutrophil recruitment could be modeled with the IVGLS to evaluate their fate within the spheroid, their cellular interaction partners, and impact on mycobacterial fitness. Different neutrophil subsets were unveiled in cattle (59), and these may variably alter features of the IVGLS or viability of mycobacteria within.

We observed that uninfected bMDM are vacuole-rich from the very beginning but accumulated lipids upon infection in IVGLS. Macrophages in the IVGLS accumulate neutral lipids, such as triacylglycerols, which are specific for foam cells in TB (60). Although the TNF-α signaling pathway may lead to foam cell formation, IVGLS do not release abundant TNF-α (60). IVGLS could be used to investigate underlying mechanisms for accumulation of lipid bodies in bovine macrophages. We did not observe a significant progression of macrophages toward polyploidy. Multinucleated giant cells were also not observed in bovine macrophage monolayers infected with BCG. Such transformed macrophages are detected in granulomas *in vivo* (61, 62). Formation of multinucleated giant cells seems largely dependent on the expression of MBP70 (63). BCG Danish was used in our study, and this BCG strain expresses negligible amounts of MBP70 due to a point mutation in SigK (64). Usage of other BCG strains and infection with MTC members could validate the reported preference of bovine cells for progression toward a polyploid phenotype in monolayers and elucidate whether the same holds true for spheroids.

The IVGLS model described is not without limitations. Although IVGLS do not fully reflect the diverse immune cell influx observed *in vivo*, they provide a platform to study the influx of pre-defined immune cell populations, e.g., lymphocytes (“mature” IVGLS). IVGLS, as described herein, do not contain tissue-resident cells, e.g., alveolar macrophages and alveolar macrophage-derived populations, or fibroblasts. These cell types could be integrated into the IVGLS model. Facilitating a prolonged incubation of the IVGLS may allow, in addition, further cell transformation, e.g., macrophage-to-myofibroblast transformation. We used high numbers of bMDM to generate large IVGLS, and the limited availability of primary cells resulted in variable sample sizes across various investigations. We studied IVGLS for 7 days, and within this time frame, factors modulating granuloma dynamics can be studied on a molecular level. These IVGLS structures do not reflect the late-stage granulomas, e.g., stage IV, with extensive accumulation of lymphocytes, necrosis, and fibrosis. Cytokines and chemokines were measured in the supernatants and may not reflect the concentrations within the IVGLS because some soluble mediators could be “trapped” within the structure and less surface area of a spheroid compared to a monolayer. We did not capture gradients of the measured mediators, and functionalized NPs may help elucidate the existence of cytokine gradients in the future. Our model needs further comparison to *in vivo* conditions. Nevertheless, congruent with the 3R principle, it is necessary to implement new advanced cell culture systems to better understand the biology of TB. Moreover, IVGLS could support vaccine and drug testing as an additional assay for ascertaining immune cell responsiveness and/or antimycobacterial activity.

We provide a three-dimensional, adjustable model of the TB granuloma, which shows a dominant type 1 immune response, foam cell transformation, as well as metabolic rewiring of the macrophages. This model supports elucidation of immunological pathways within TB granulomas over time, including zonation and immune topography. IVGLS could contribute to vaccine development for cattle, whereas unveiling resistance mechanisms may help devise novel interventions for human TB.

## SUPPLEMENTAL MATERIAL

Supplemental figures msphere.00595-25-s0001.pdf Fig. S1-S8.

Legends msphere.00595-25-s0002.docx Supplemental figure and video legends.

Table S1 msphere.00595-25-s0003.xlsx Raw data referring to Fig. 6.

Video S1 msphere.00595-25-s0004.avi Cell viability assessment of IVGLS at 3 dpi.

ASM does not own the copyrights to Supplemental Material that may be linked to, or accessed through, an article. The authors have granted ASM a non-exclusive, world-wide license to publish the Supplemental Material files. Please contact the corresponding author directly for reuse.
