# Supplementary material for: Modeling immune responses of cattle to Mycobacterium bovis using magnetic bioprinted granulomas
Source: mSphere. 2025 Oct 31;10(11):e00595-25. doi: 10.1128/msphere.00595-25 (PMC12645956; doi:10.1128/msphere.00595-25)
Supplement: Supplemental figures — Fig. S1-S8. [file msphere.00595-25-s0001.pdf]

Figure S1

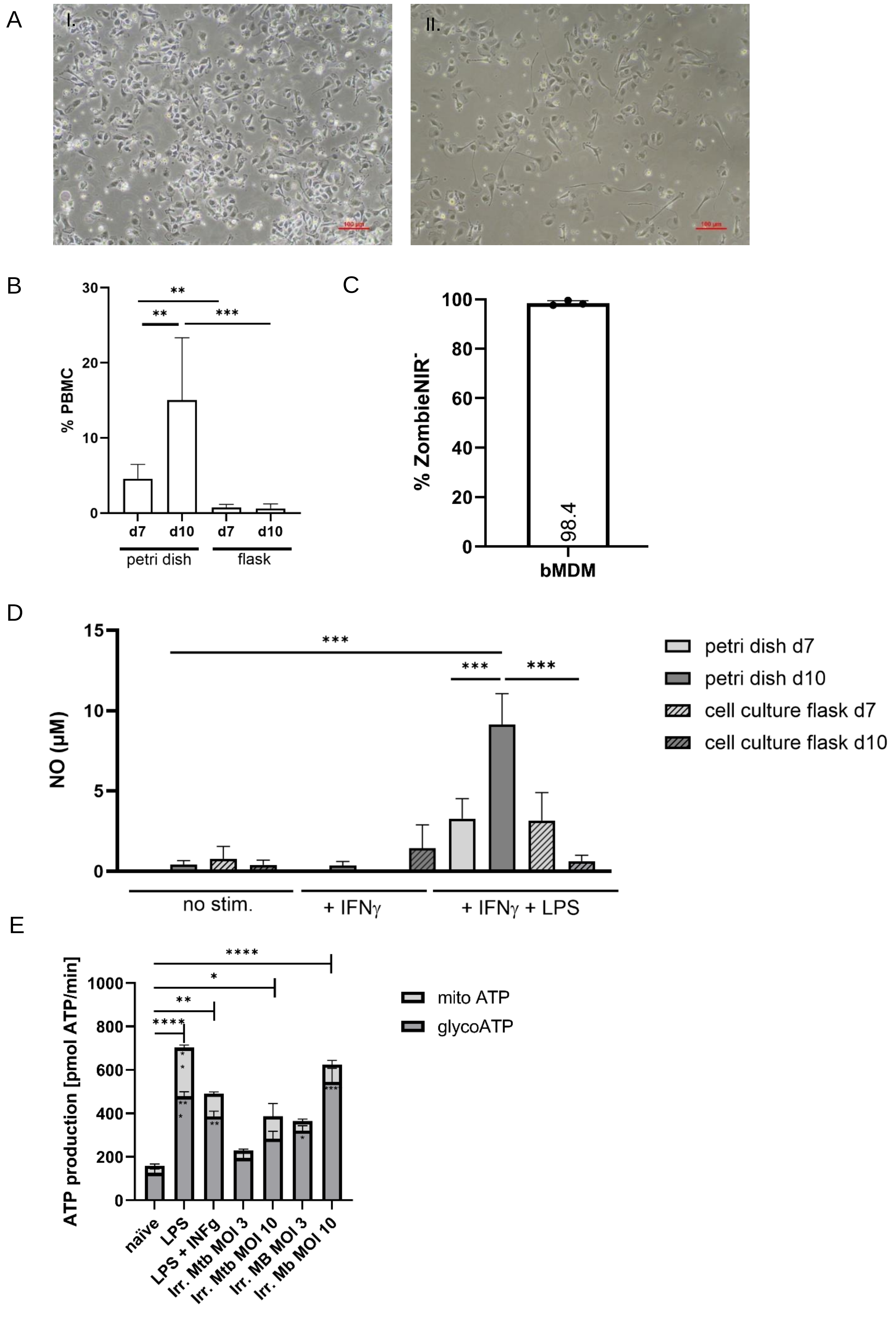

A
I.
II.
B
% PBMC
30
20
10
0
**
**
***
d7
d10
d7
d10
petri dish
flask
C
% ZombieNIR⁻
100
80
60
40
20
0
98.4
bMDM
D
NO (µM)
15
10
5
0
***
***
***
petri dish d7
petri dish d10
cell culture flask d7
cell culture flask d10
no stim.
+ IFNγ
+ IFNγ + LPS
E
ATP production [pmol ATP/min]
1000
800
600
400
200
0
****
*
**
****
mito ATP
glycoATP
naïve
LPS
LPS + INFg
Irr. Mtb MOI 3
Irr. Mtb MOI 10
Irr. MB MOI 3
Irr. Mb MOI 10

Figure S2

A

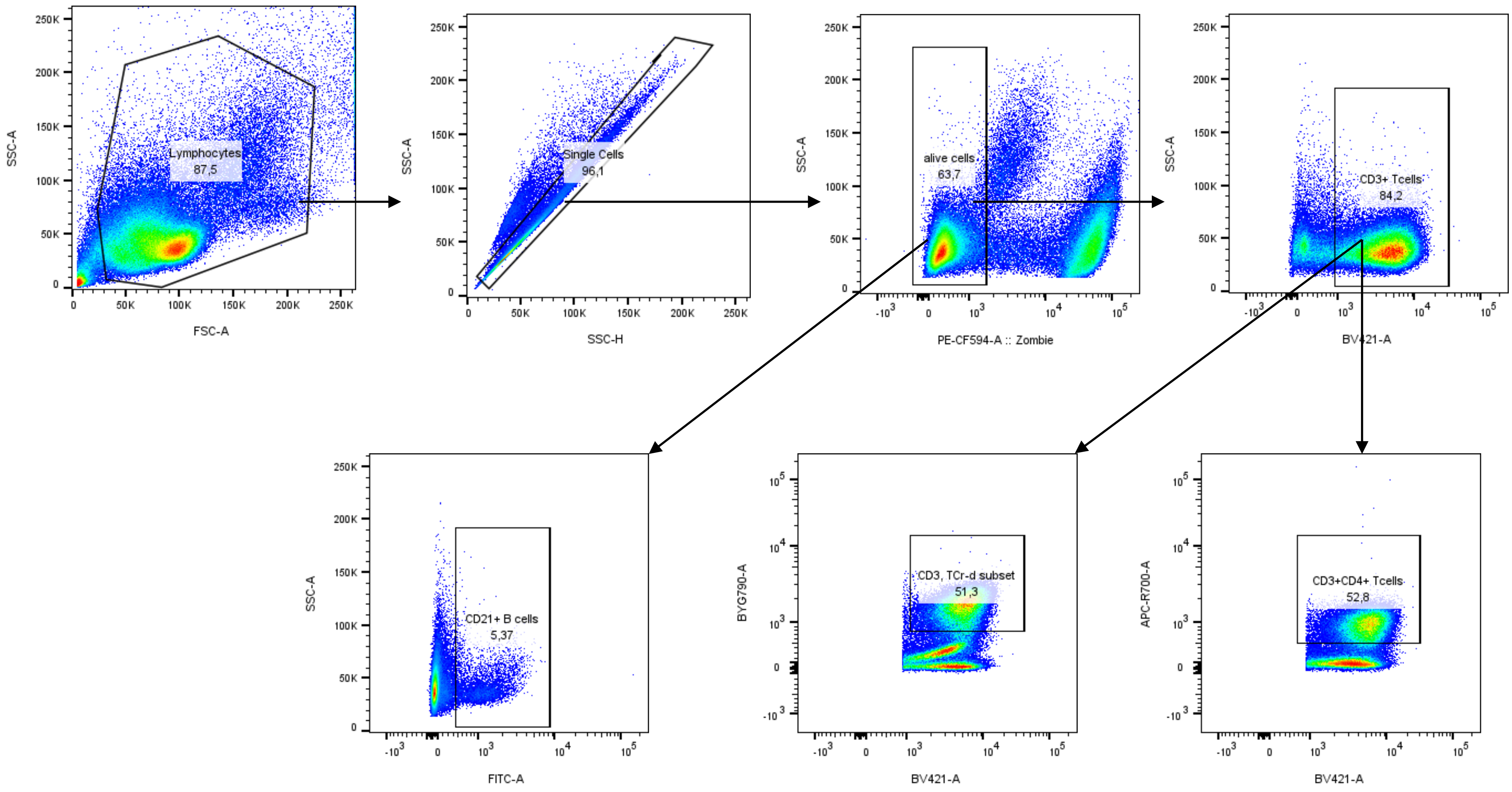

B

of alive lymphocytes	Animal 1	Animal 2	Animal 3	Animal 4
%CD3$^+$	79.7	77.2	85.7	84.2
%CD21$^+$	3.65	7.62	4.82	5.37
of CD3+				
%CD4$^+$CD3$^+$	22.8	50.0	25.1	52.8
%TCR-d$^+$CD3$^+$	22.9	52.1	34.9	51.3

Figure S3

A

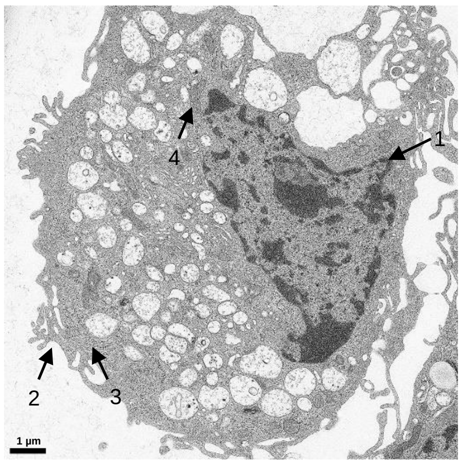

B

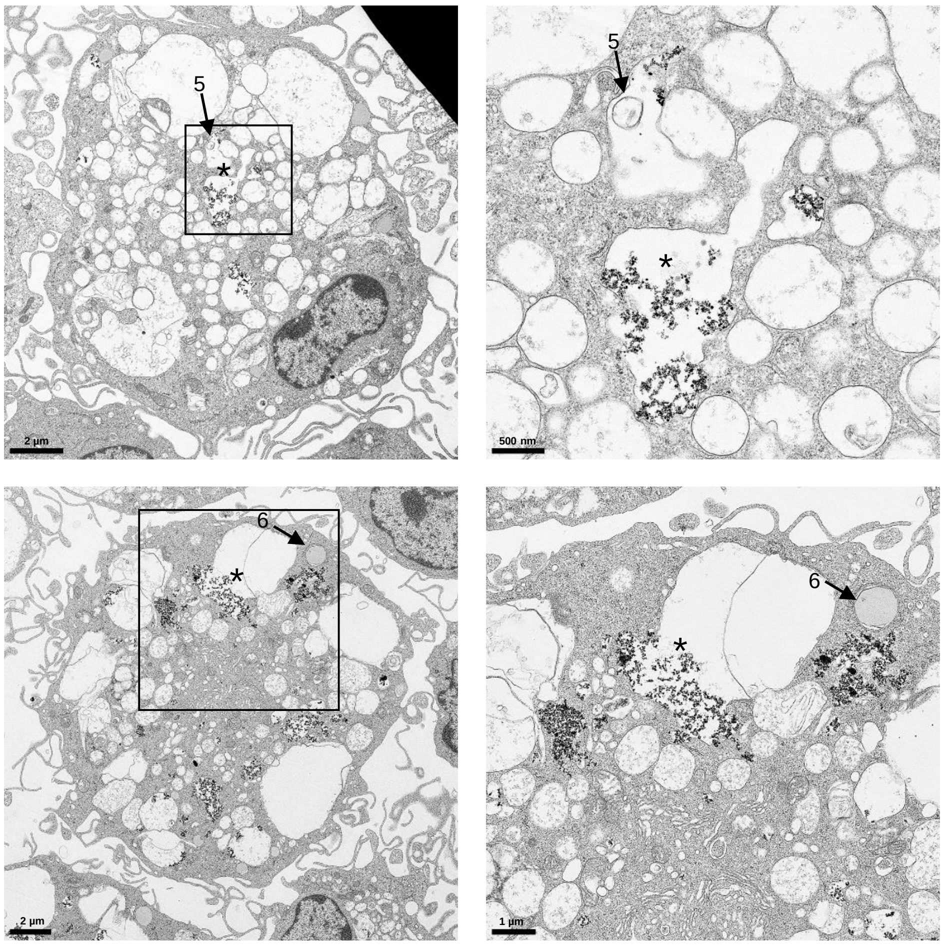

Figure S4

A

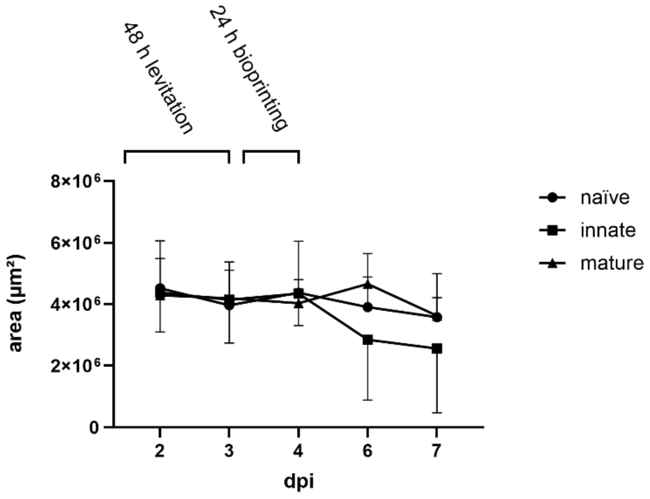
48 h levitation
24 h bioprinting
8×10⁶
6×10⁶
4×10⁶
2×10⁶
0
area (µm²)
2
3
4
6
7
dpi
naïve
innate
mature

B

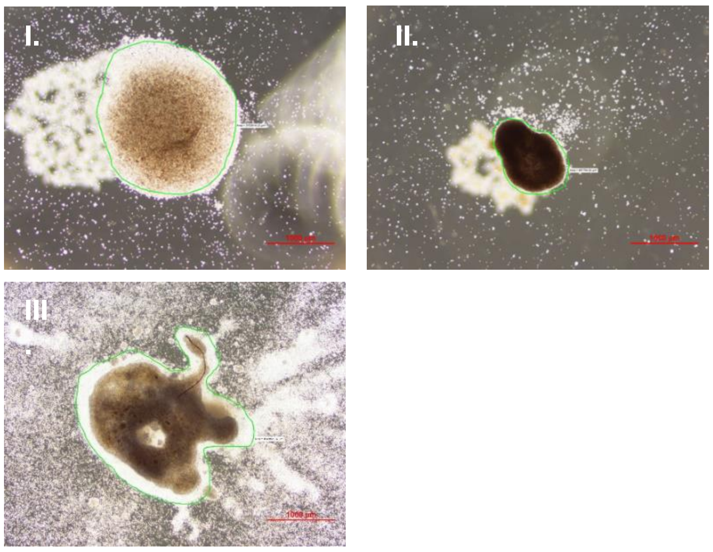
I.
II.
III.
1000 µm
1000 µm
1000 µm

Figure S5

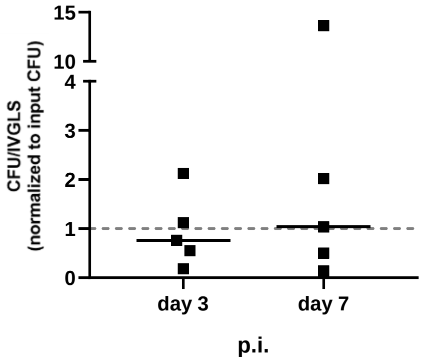

Figure S6

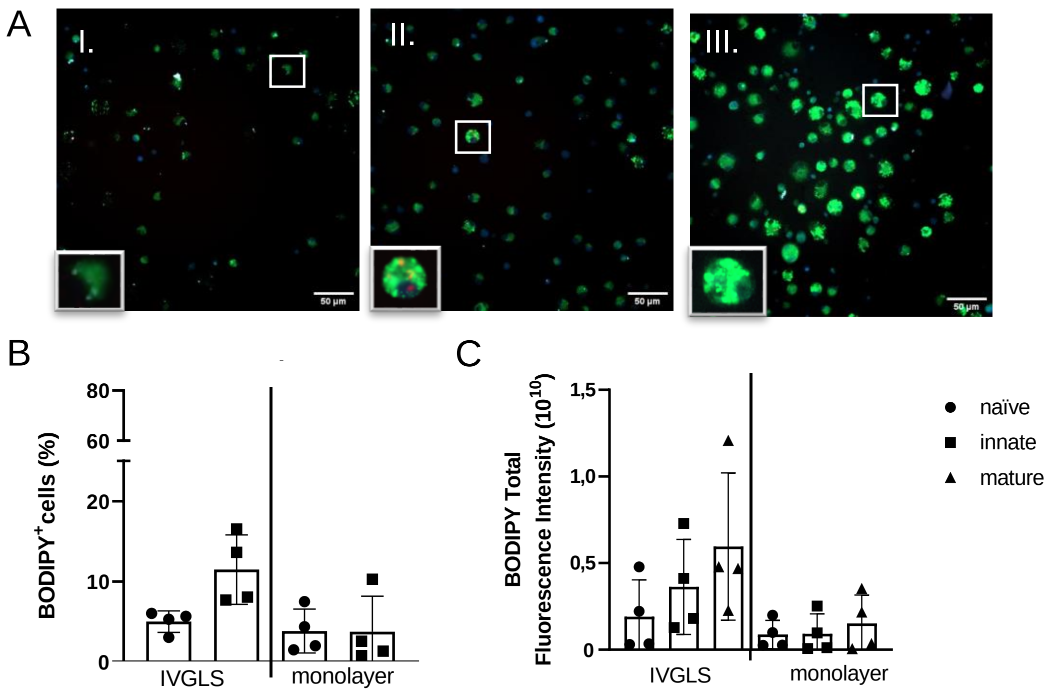
A
I.
II.
III.
50 µm
B
BODIPY+cells (%)
80
60
20
10
0
IVGLS
monolayer
C
BODIPY Total
Fluorescence Intensity (10^10)
1,5
1,0
0,5
0
IVGLS
monolayer
naïve
innate
mature

Figure S7

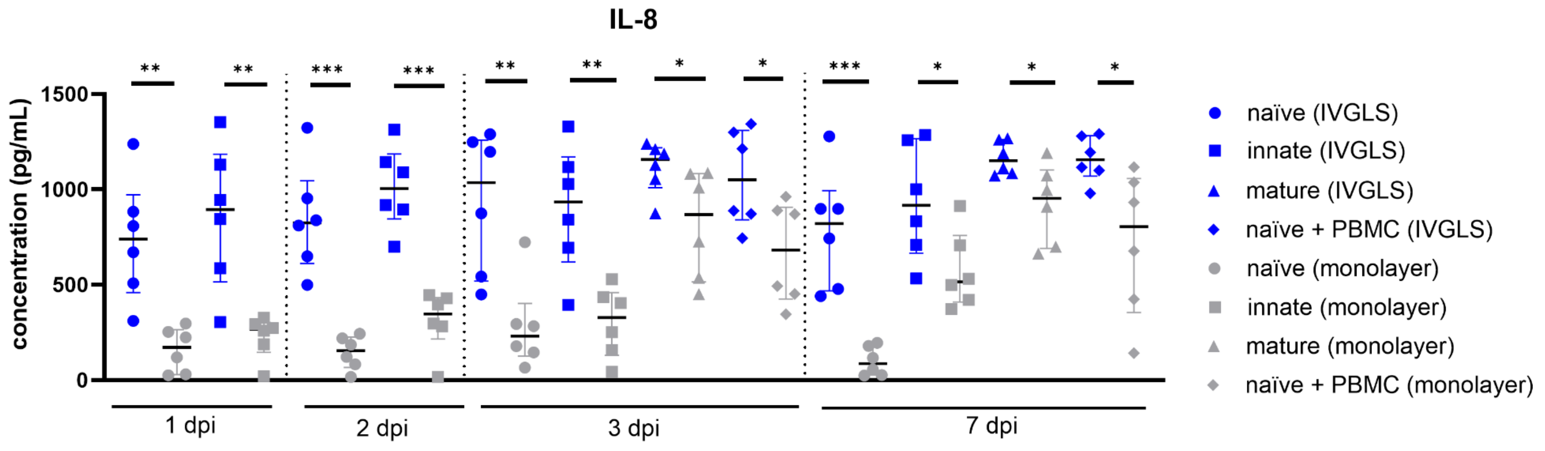

IL-8
concentration (pg/mL)
1500
1000
500
0
**
***
*
1 dpi
2 dpi
3 dpi
7 dpi
naïve (IVGLS)
innate (IVGLS)
mature (IVGLS)
naïve + PBMC (IVGLS)
naïve (monolayer)
innate (monolayer)
mature (monolayer)
naïve + PBMC (monolayer)

Figure S8

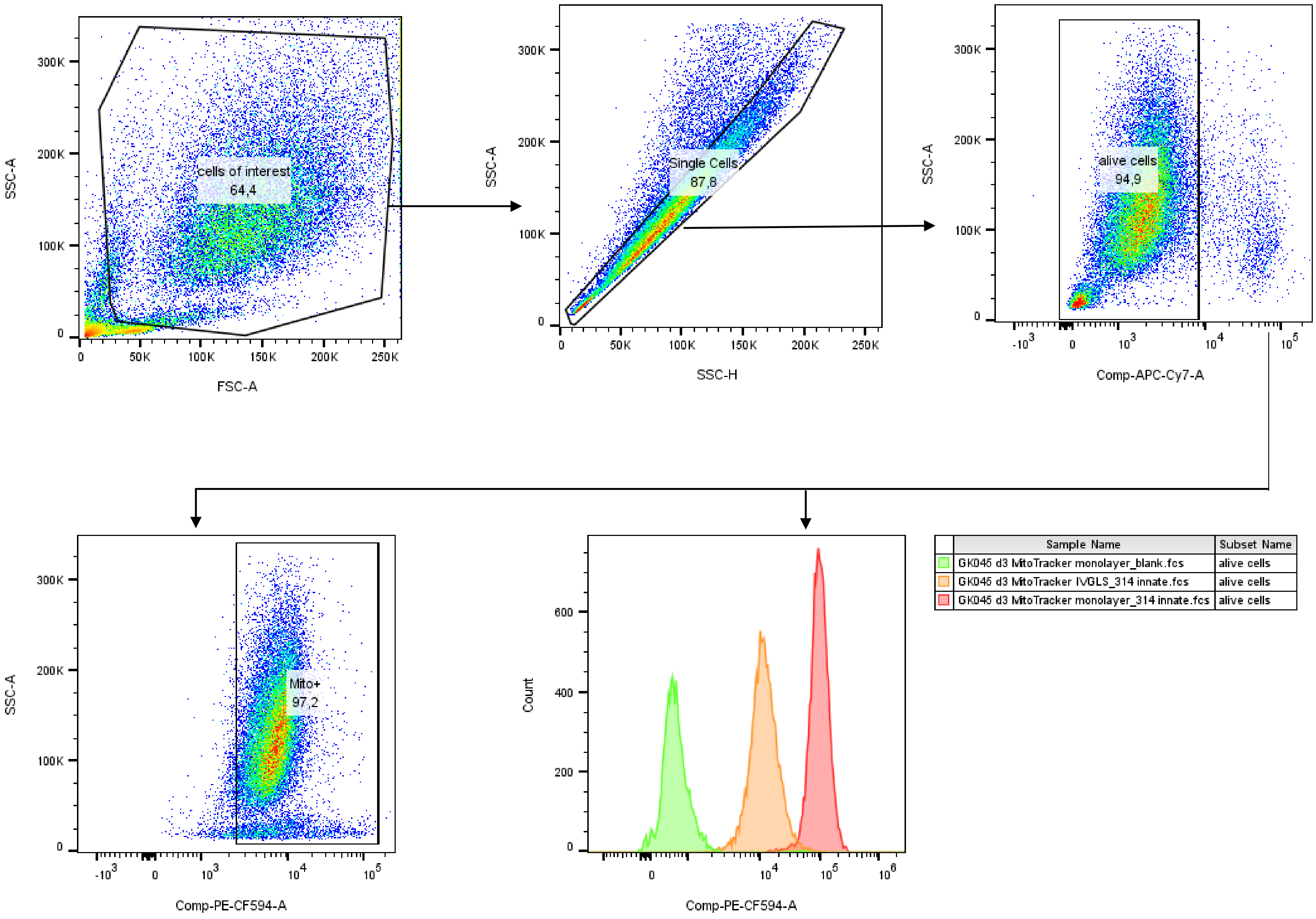
cells of interest
64,4
SSC-A
FSC-A
Single Cells
87,8
SSC-H
alive cells
94,9
Comp-APC-Cy7-A
Mito+
97,2
Comp-PE-CF594-A
Count
Sample Name
Subset Name
GK045 d3 MitoTracker monolayer_blank.fcs
alive cells
GK045 d3 MitoTracker IVGLS_314 innate.fcs
alive cells
GK045 d3 MitoTracker monolayer_314 innate.fcs
alive cells
