# Supplementary material for: Modeling immune responses of cattle to Mycobacterium bovis using magnetic bioprinted granulomas
Source: mSphere. 2025 Oct 31;10(11):e00595-25. doi: 10.1128/msphere.00595-25 (PMC12645956; doi:10.1128/msphere.00595-25)
Supplement: Legends — Supplemental figure and video legends. [file msphere.00595-25-s0002.docx]

**Supplementary Figure Legends**

mSphere00595-25

**Figure S1. Features of bovine monocyte-derived macrophages differentiated using different culture substrates.**

(A) Microscopic images of bovine monocyte-derived macrophages (bMDM) cultured for 7 days in Petri dishes (I.) and cell culture flask (II.) Representative images show morphologic features of MDM and cell density. (B) Yield of bMDM given as percentage of bMDM of PBMC seeded. Monocytes were differentiated for 7 or 10 days (d7, d10) in Petri dishes or cell culture flasks, respectively (Petri dish 7 days: n=9, Petri dish 10 days: n=11, cell culture flask 7 days: n=3, cell culture flask 10 days: n=9). Data shows mean ± SD, one-way ANOVA with Tuckey’s post-hoc test. (C) Viability of bMDMs cultured in Petri dishes for 7 days assessed by flow cytometry. After differentiation bMDMs were detached, stained with ZombieNIR dye and viability was measured by flow cytometry. Viable cells defined as ZombieNIR^-^ were adjusted to FMO, (n=3) (D) Nitric oxide (NO) production in bMDM cultured for 7 days in Petri dishes (light grey bar; n=9), cultured for 10 days in Petri dishes (dark grey bar; n=9) and cultured for 7 and 10 days in cell cultures flasks (striped bar; n=4-5) and stimulated as indicated. Each data point represents a biological replicate, measured as triplicates. Statistical analysis was by one-way ANOVA with Tuckey’s post-hoc test. (E) Metabolic flux analysis of naïve and stimulated bMDM. Total ATP production and contribution of glycolytic ATP (glucoATP) and mitochondrial ATP (mitoATP) in bMDM are shown. Data from one representative experiment out of three is shown, with 3-5 technical replicates for each condition. ATP depicted as mean ± SEM, *p < 0.05, **p < 0.01, ***p < 0.001, ****p < 0.0001, one-way ANOVA with Dunnett’s test for multiple comparisons. Significance on top indicates changes in total ATP production, changes in contributing glycolytic or mitochondrial production are indicated within bars compared to naive.

**Figure S2. Frequencies of lymphocyte populations in autologous PBMC fraction.**

(A) Dot plots depict the gating strategy of bovine B cells and different subsets of T cells from autologous non-adherent PBMCs gained during monocyte isolation. Gates were set according to FMO controls. (B) Percentages of B cells and different subsets of T cells of alive lymphocytes. Data of the four cattle used as blood donors are shown.

**Figure S3. Ultrastructure of bMDM labelled with NPs**

(A) naive bMDMs without NPs (B) bMDMs labelled with NPs. Rectangle marks region of interest (ROI). Arrows indicate cell components: 1- Nucleus with heterochromatin (dark) and euchromatin, 2-pseudopodia, 3- rough endoplasmic reticulum, 4- mitochondria; 5- membrane residues; 6- lipid bodies. Stars mark cluster of NPs. Black bar shows indicated scale.

**Figure S4. Prolonged levitation period does not alter dynamics of IVGLS generation.**

(A) Area in µm² of naïve (grey), innate (blue) and mature (orange) IVGLS over time. Data shows mean ± SD. (B) (I.) Naïve, (II.) innate, and (III.) mature IVGLS shown with 2x magnification. Green line indicates area measurements. Scale bar corresponds to 1 mm.

**Figure S5. BCG burdens in monolayers of bMDM.**

CFU from cell lysates of bMDM cultured as monolayer. Data was normalized to bacterial uptake at 4 h post infection (grey dotted line).

**Figure S6. Cell transformation to foamy cells evaluated by high-content imaging.**

(A) Images show lipid load in dislodged macrophages from (I.) naïve, (II.) innate and (III.) mature IVGLS. Images show nuclei (blue), BCG mCherry (red) and lipid bodies (BODIPY 493/503; green), scale bar 50 µm. Overlays done with HCS Systems software. (B) Percentage of BODIPY positive cells from dislodged naïve () and innate (rectangle) IVGLS and respective monolayer specimens acquired using high-content imaging at 3 dpi (n=4). (C) Total fluorescence intensity of BODIPY 493/503 from dislodged naïve, innate, mature IVGLS or monolayer at 3 dpi (n=4). A minimum of 1000 cells per spheroid were evaluated.

**Figure S7. IL-8 concentrations in various IVGLS over time.**

IL-8 was measured by ELISA in cell-free supernatants from IVGLS (blue) and monolayers (grey). Naïve, innate and mature IVGLS as well as naïve macrophages with PBMC (rhombus). Autologous PBMC were added 2 dpi. Statistical analyses done by unpaired, two tailed t-test (n=6), p < 0.05 * p< 0.01 ** p < 0.001 ***.

**Figure S8. Gating strategy for analysis of mitochondria (MitoTracker orange) in bMDM.**

Flow cytometry dot plots show the gating strategy, which included gating on myeloid cells, singlets, life cells. The MitoTracker orange-positive gate was adjusted using FMO controls. Histogram shows mean fluorescence intensities (MFI) of MitoTracker orange in myeloid cells from monolayer (red) and IVGLS (blue).

**Supplementary Video 1. Cell viability assessment of IVGLS at 3 dpi.**

Video shows innate IVGLS 3 dpi stained with Hoechst 34580 (blue, nucleus), Caspase 3/7 Dye (green, apoptotic cells) and BCGmCherry (red). Image acquisition was done with the instant computational clearing mode within the Leica Application Suite X software. Step size was 3.80 µm, number of steps was adjusted to the depth of IVGLS. After 3D rendering, the video was recorded and saved using high-quality render settings.
